# Clinical correlates of parametric digit-symbol substitution test in schizophrenia

**DOI:** 10.1016/j.ajp.2014.03.010

**Published:** 2014-08

**Authors:** Anekal C. Amaresha, Vijay Danivas, Venkataram Shivakumar, Sri Mahavir Agarwal, Sunil V. Kalmady, Janardhanan C. Narayanaswamy, Ganesan Venkatasubramanian

**Affiliations:** a Department of Psychiatry, National Institute of Mental Health & Neurosciences (NIMHANS), Bangalore, India; b Translational Psychiatry Laboratory, Cognitive Neurobiology Division, Neurobiology Research Centre, National Institute of Mental Health & Neurosciences, Bangalore, India

**Keywords:** Schizophrenia, Processing speed, Cognitive deficits, Negative symptoms

## Abstract

Processing speed deficit, ascertained by digit-symbol substitution test (DSST), is considered as a fundamental impairment in schizophrenia. Clinical correlates of processing speed abnormalities, especially using the parametric version of DSST is yet to be evaluated comprehensively. In this study, we examined schizophrenia patients (*N* = 66) and demographically matched healthy controls (*N* = 72) using computer-administered parametric DSST (pDSST) with fixed (pDSST_F_) as well as random (pDSST_R_) conditions and analysed the relationship between pDSST performance and clinical symptoms. Psychopathology was assessed using Scale for Assessment of Positive Symptoms (SAPS)/Negative Symptoms (SANS) with good inter-rater reliability. In comparison with healthy controls, patients demonstrated significantly lesser number of correct responses (*C*_N_) in pDSST_F_ (*t* = 8.0; *p* < 0.001) and pDSST_R_ (*t* = 7.8; *p* < 0.001) as well as significantly prolonged reaction time in pDSST_F_ (*t* = 7.1; *p* < 0.001) and pDSST_R_ (*t* = 7.0; *p* < 0.001). The difference in *C*_N_ between pDSST_F_ and pDSST_R_ [Δ*C*_N_] was significantly lesser in patients than healthy controls (*t* = 2.61; *p* = 0.01). The pDSST reaction time had significant positive correlation with negative syndrome scores as well as bizarre behaviour score. Significantly greater processing speed deficits in pDSST suggest potential relational memory/visual scanning abnormalities in schizophrenia. Furthermore, pDSST deficits demonstrated a significant association with the psychopathology, especially with the various negative symptoms and bizarre behaviour.

## 1 Introduction

Schizophrenia is a psychiatric disorder associated with marked disability. The predominant symptoms of this disorder can be categorised as positive symptoms like delusions and hallucinations, and negative symptoms like affective flattening, anhedonia, alogia and apathy (Mueser and McGurk, 2004). In addition, cognitive symptoms are increasingly being recognised as an integral part of the illness with a close link to the pathophysiological aspects of this illness (Harvey, 2012). Cognitive deficits in schizophrenia have been associated with disorganisation and negative symptoms (Cohen et al., 1999, Green et al., 2000, Kerns and Berenbaum, 2002) as well as with poor functional outcomes (Green, 1996, Green, 1998, Weinberger and Gallhofer, 1997). Deficits in terms of attention, processing speed, memory, working memory, and executive function have all been consistently reported in patients with schizophrenia (Reichenberg and Harvey, 2007).

Recent meta-analyses of specific cognitive domains in schizophrenia have demonstrated that processing speed deficit as a fundamental impairment in this disorder (Dickinson et al., 2007, Henry and Crawford, 2005). Processing speed, which reflects the speed with which different cognitive operations can be executed (Dickinson et al., 2007), is usually quantified by the measuring the number of successful completion of trials of a simple task by a subject over a brief time period. A classical task that is used to assess processing speed is the Digit Symbol Substitution Test (DSST); in this task, the subject is instructed to utilise information from digit-symbol pairing to deduce the key for symbols associated with a digit series over a specific time (Dickinson et al., 2007). Various cognitive components that are tested in this task comprise of scanning, matching, switching, and writing operations that are reflective of several higher cognitive functions like perception, encoding and retrieval processes, transformation of information stored in active memory and decision making (Salthouse, 1996).

In schizophrenia, processing speed as measured by the digit symbol substitution task is considered to be a critically impaired cognitive ability (Dickinson et al., 2007, Henry and Crawford, 2005, Knowles et al., 2010). This aspect is further underscored by the demonstrated relationship of processing speed to the illness risk in schizophrenia (Glahn et al., 2007, Niendam et al., 2003, Reichenberg et al., 2010), severity of illness (Dickinson et al., 2007) and the disability due to the illness (Brekke et al., 2007). Moreover, the processing speed, as examined by a digit symbol coding task, was shown to be severely affected in even in first-episode psychosis (Gonzalez-Blanch et al., 2007). Also, deficits in processing speed might mediate a wide range of other cognitive deficits (Rodriguez-Sanchez et al., 2007).

Recently, a parametric Digit Symbol Substitution Test (pDSST) with manipulation of demands placed on visual scanning efficiency and relational memory while keeping decisional and motor requirements constant was shown to be useful in demonstrating the potential relational memory deficits that underlie the digit-symbol coding abnormalities in schizophrenia (Bachman et al., 2010). Given the shared neural basis for fronto-hippocampal deficits for both the relational memory (Hanlon et al., 2012) abnormalities as well as negative symptoms of schizophrenia (Potkin et al., 2002) as well as the fact that the major cognitive deficits and negative symptoms are mediated and explained through “hypofrontality” (Lesh et al., 2011, Lin et al., 2013, O’Leary et al., 2000), it is tempting to hypothesise that pDSST task deficits will correlate with negative symptoms in these patients. With this hypothesis, in this study, we evaluated the processing speed using computerised pDSST in subjects with DSM-IV schizophrenia in comparison with matched healthy controls (HC) as well as the relationship of this task performance with clinical symptom scores.

## 2 Methods

### 2.1 Subjects

Patients attending the clinical services of the National Institute of Mental Health & Neurosciences (India), who fulfilled DSM-IV criteria (APA, 1994) for schizophrenia (*N* = 66), were examined in this study. The diagnosis of schizophrenia was established using Mini International Neuropsychiatric Interview (MINI) Plus (Sheehan et al., 1998), which was confirmed by another psychiatrist through an independent clinical interview. The details related to illness onset and antipsychotic-naïve status were carefully ascertained by reliable information obtained from at least one reliable adult relative. Clinical symptoms were assessed using Scale for Assessment of Positive Symptoms (SAPS) (Andreasen, 1984) and Scale for Assessment of Negative Symptoms (SANS) (Andreasen, 1983) with good inter-rater reliability. The antipsychotic dose was converted to chlorpromazine equivalents based on standard conversion principles (Woods, 2003).

Healthy controls (*N* = 72), who volunteered for study, were screened to rule out any psychiatric diagnosis using the MINI plus as well as a comprehensive mental status examination. None of the controls had family history of psychiatric disorder in first-degree relatives. Patients and controls did not have features suggestive of alcohol abuse/dependence. None had history or clinical feature suggestive of neurological/medical disorder. None had any developmental delays or mental retardation. After complete description of study to the subjects, written informed consent was obtained. The Institute's ethics committee approved the study.

### 2.2 Parametric DSST task

**Fig. 1:**
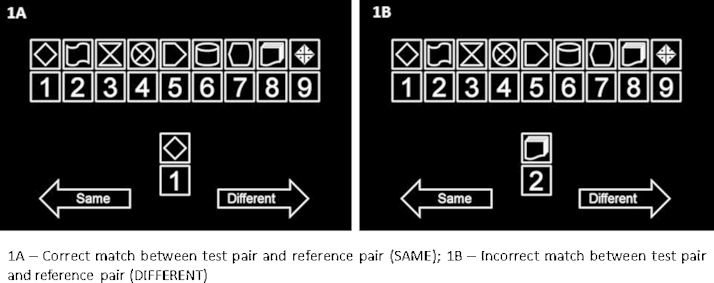
shows representative test stimuli of parametric digit-symbol substitution.

This study adopted the parametric digit symbol coding task (pDSST), as reported by a recent study (Bachman et al., 2010). Computerised version of this task was implemented using E-Prime 2.0 professional version (www.pstnet.com; Psychology Software Tools, Inc., PA, USA). The task was administered in a quiet environment with subjects being seated at about 50 cm from the computer screen. The pDSST comprised of two conditions–namely, fixed and random, during which the set size and presentation consistency are varied over 20-s blocks of trials. The efficiency of visual scanning as well relational memory were tested by randomly changing the number of digit symbol pairs from 3 to 6 to 9; this, in turn, changes the demands placed upon both scanning efficiency and relational memory. On the contrary, limits of relational memory were assessed by maintaining the digit symbol pairing throughout the block of trials (Fixed Condition) or by randomly assigning these pairing for each trial in a 20-s block (Random Condition). The subjects were shown a main reference set of digits and symbols in pairs and a test set which is a single set of digit symbol pair under the main reference set. The subjects were instructed to compare the test pair with the reference set of pairs and indicate whether the test pair is same or different from that of the depicted pairing in the reference set by pressing a pre-specified button (left or right arrow key respectively as indicated in Fig. 1) in the computer keyboard. All subjects underwent a practice trial comprising of five trials of varying number of reference digit symbol pairs to familiarise themselves with the experiment.

Thus, the pDSST had two conditions called fixed and random (conditions) and lasted for four minutes. Each condition had three types of reference sets with 3, 6 and 9 digit symbol pairs. The task had a total of 12 blocks of trials (6 types of blocks – 3 in random and 3 in fixed, repeated twice in a random order) and each block lasted for 20 s duration. During the task, the accuracy and reaction time were recorded for both fixed and random conditions on all the 3 reference set types.

The fixed condition was predictable if respondents kept the reference set in their mind. However, the reference set increases to 6 or 9 digit symbol pairs, it would be challenging for their immediate or relational memory since it hampers the performance in terms of accuracy and reaction time. On the other hand, the digit symbol pairings in the random condition were changed constantly for each reference set. Hence, for the better performance one should have good visual scanning to compare the targeted digit symbol pairs.

## 3 Statistical analysis

Statistical Package for Social Sciences (SPSS-13) was used for analysis. Data was assessed using Shapiro–Wilk test and was found to be normally distributed. Independent samples *t*-test and chi-square test were used for continuous and discrete variables for comparisons. We employed partial correlation to examine the relationship between pDSST variables and the illness severity scores after controlling for the confounding effects of years of education.

## 4 Results

Schizophrenia patients and healthy controls did not differ significantly in age, sex ratio and years of education (*p* > 0.05; Table 1). As shown in Table 2, the patients had a significantly poorer performance in all the pDSST measures compared to HC.

**Table 1:** Profile of clinical variables in schizophrenia patients and healthy controls.

Variable	Patients (*N* = 66)	Controls (*N* = 72)	Statistic^a^	*p*
	Mean ± SD	Mean ± SD		
Age (years)	29.7 ± 6.2	28.4 ± 6.2	*t* = 1.2	0.2
Sex ratio (men:women)	34:32	46:26	*χ*^2^ = 0.1	0.2
Education score	5.0 ± 1.5	4.8 ± 1.4	*t* = 0.6	0.5
Total SAPS score	22.4 ± 21.1	–	–	–
Total SANS score	38.3 ± 29.1	–	–	–
Chlorpromazine equivalents	435.7 ± 268.0	–	–	–

^a^*t* = independent samples *t*-test; *χ*^2^ = chi-square statistic.

**Table 2:** Comparative profile of parametric digit symbol substitution task performance between schizophrenia patients and healthy controls.

Variable	Patients (*N* = 66)	Controls (*N* = 72)	*t*^a^	*p*
	Mean ± SD	Mean ± SD		
Number of correct responses (fixed condition)	38.2 ± 12.4	57.1 ± 15.2	8.0	<0.001
Number of correct responses (random condition)	36.4 ± 11.3	53.2 ± 13.6	7.8	<0.001
Reaction time (fixed condition) [s]	3.4 ± 1.2	2.3 ± 0.7	7.1	<0.001
Reaction time (random condition) [s]	3.7 ± 1.2	2.5 ± 0.7	7.0	<0.001

^a^Independent samples *t*-test.

Difference between the number of correct responses during the fixed and random conditions [Δ*C*_N_ = fixed minus random] was significantly lesser in schizophrenia patients (1.8 ± 4.4) in comparison with HC (4.0 ± 5.2) [*t* = 2.6; *p* = 0.01].

**Fig. 2:**
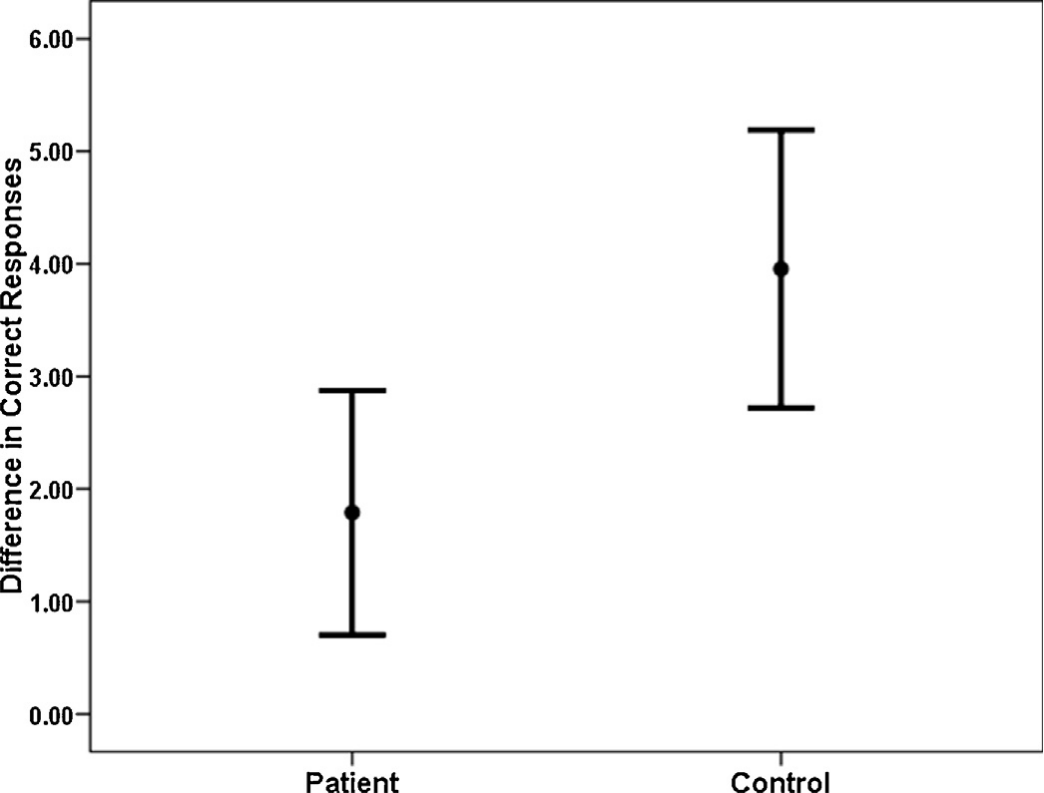
Comparative profile of difference in correct responses (fixed minus random conditions) during the parametric DSST: schizophrenia patients (*N* = 66) vs. healthy controls (*N* = 72).

**Fig. 3:**
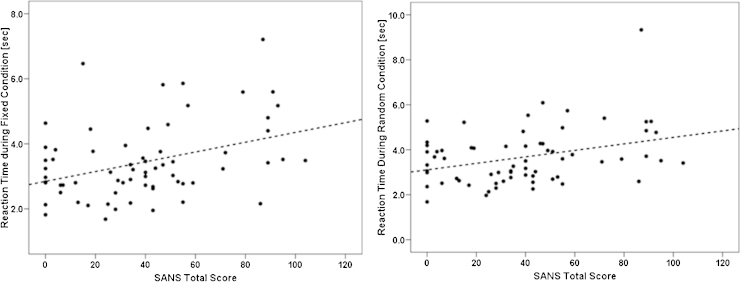
Significant positive correlation between reaction time during parametric DSST and negative syndrome score in schizophrenia patients (*N* = 66).

Years of education had a significant positive correlation with majority of the pDSST parameters of interest (*p* ≤ 0.01) among the patients with schizophrenia as well as HC. Hence, years of education were controlled for in the partial correlation analysis between SANS/SAPS scores and pDSST parameters for the patients with schizophrenia. There was significant positive correlation for pDSST fixed condition reaction time with total SANS score (*r* = 0.38; *p* = 0.001), alogia score (*r* = 0.36; *p* = 0.003), inattention score (*r* = 0.43; *p* = 0.001), affective flattening score (*r* = 0.35; *p* = 0.004) and SAPS bizarre behaviour score (*r* = 0.39; *p* = 0.001). Also, pDSST random reaction time correlated significantly with SANS total score (*r* = 0.35; *p* = 0.004), inattention score (*r* = 0.36; *p* = 0.003) and SAPS bizarre behaviour score (*r* = 0.36; *p* = 0.004). The relationship of total SANS score with pDSST fixed reaction time and pDSST random reaction time is depicted in Fig. 2. No significant correlation was observed between the chlorpromazine equivalents and any of the pDSST parameters in schizophrenia patients (Fig. 3).

## 5 Discussion

Our study findings suggest schizophrenia patients to have significantly greater processing deficits in pDSST compared to the HC. Furthermore, these deficits had a significant association with the psychopathology, especially the various negative symptom sub-score and bizarre behaviour. The patients with schizophrenia performed poorly compared to HC in all the measures of the pDSST including the accuracy and reaction time. This was observed in both the fixed and random conditions. Fixed and random conditions test relational memory and visual scanning respectively (Bachman et al., 2010). Thus, the patients with schizophrenia demonstrate deficits in both these cognitive modalities in comparison to HC. Additionally, the difference in the correct responses between fixed and random conditions [Δ*C*_N_] was significantly lesser among the patients compared to HC which is in tune with the previous observation (Bachman et al., 2010). This indicates possible relational memory impairment in schizophrenia patients. Memory deficits in schizophrenia are selective and impairments of associative/relational memory are well known (Achim and Lepage, 2003, Armstrong et al., 2012). This cognitive faculty needs the binding together of disparate memory elements and it plays a critical role in information processing speed. Our finding, thus, substantiates the previous findings of restriction in relational memory as an imperative factor for reduced processing speed (Bachman et al., 2010).

Relational memory has been linked somewhat convincingly to the integrity of hippocampal functioning (Keri et al., 2012, Ongur et al., 2006). Various lines of evidences suggest hippocampus structural (Wright et al., 2000) and functional impairment (Ledoux et al., 2013) in schizophrenia. Hippocampal volume and shape abnormalities have been documented in schizophrenia (McClure et al., 2013). Hippocampal changes could present with wide implications in the cognitive underpinnings of psychosis; however, the present evidence favours a greater emphasis on relational memory impairment. In this context, the present finding of very prominent relational memory impairment as demonstrated by this sensitive test needs attention.

An alternate explanation to this significant deficit in processing speed comes from the fact that higher order cognitive processes need an intricate interaction between many critical brain regions. Even though a greater emphasis is placed on the role of hippocampus, anomalous interaction between hippocampus and various brain regions like prefrontal cortex, basal ganglia and cerebellum could underlie the cognitive pathology. Disturbed hippocampal–prefrontal interactions have been demonstrated in individuals during early psychosis as well as for those at risk of developing psychosis (Benetti et al., 2009). Indeed, decreased fronto-temporal anatomical connectivity measured through white matter anatomical integrity of uncinate fasciculus has been shown to be associated with deficient relational memory performance in schizophrenia (Hanlon et al., 2012).

A meticulous coordination of various cognitive components like memory encoding and retrieval, visual scanning and matching, response selection and execution which are integral components of the task examining the speed of processing appears to be essential. This could take a longer time than needed if any of the sub-components is affected or if the various components are not co-ordinated. There is emerging evidence for schizophrenia as a disorder of neural dysconnectivity (Schmitt et al., 2011) indicating that the cognitive coordination could be disrupted, resulting in deficits in processing speed.

What is more interesting is the relationship between the processing speed parameters and the psychopathology scores. The current study demonstrates a robustly significant positive relationship between fixed condition reaction time and negative symptoms of affective flattening, alogia and inattention among the patients with schizophrenia. Similarly, the random condition reaction time had a significant positive correlation with inattention. This observation is in support of the earlier finding relating processing speed deficit with negative syndrome in schizophrenia (McDowd et al., 2011).

Processing speed has been found to be an important cognitive domain that has been related most consistently to the level of functioning and quality of life among schizophrenia patients (Aksaray et al., 2002, Matsui et al., 2008). Negative symptoms have been considered to be integral to the pathology of schizophrenia and they also are closely linked to the functional outcome (Rabinowitz et al., 2012). The relation between negative symptoms and speed of processing appears to be complex and is not completely understood. However, the hypofrontality theory of schizophrenia identifies common brain substrate for both these factors, even though the cause–effect relationship between these factors is still obscure (Penades et al., 2002). The intricate relationship and correlation between these two parameters have been reported recently in schizophrenia (McDowd et al., 2011, Ojeda et al., 2012). In line with these previous observations, our study also demonstrates the relationship between negative symptoms and processing speed. Impaired relational memory as well as the visual scanning subcomponents of the task correlated with greater negative symptoms. Considering differential substrates for these two sub-tasks, one could argue that altered connectivity between frontal and other regions as discussed previously might be responsible for this relationship. Altogether, this underscores the importance of combined measures to treat this cognitive deficit and negative symptoms. The cognitive remediation strategies need to target processing speed as a major component of intervention.

The reason for the relationship between bizarre behaviour and processing speed deficits is unclear. In a previous study examining the clinical correlates of cognitive impairments, it was observed that disorganisation and reality distortion have correlation with cognitive deficits including processing speed deficits (Galderisi et al., 2009). Also, as shown in another study, disorganisation had a discernible effect on the ability to quickly detect an expected stimulus and discriminate a valid stimulus from an invalid one, a cognitive faculty which requires visual scanning and attention (Amado and Olie, 2006). The bizarre behaviour sub-score of SAPS includes items which could reflect disorganisation to some extent.

One of the study limitations is that formal IQ assessment has not been done. However, no subjects had history of delay in developmental milestones and there was no clinical evidence for mental sub-normality in any subjects. In addition, the years of formal education is comparable between the two groups. Examining antipsychotic-treated schizophrenia patients might be construed as another potential limitation. However, we did not find any significant correlation between antipsychotic dose and pDSST parameters in patients suggesting that medications might not have contributed to the sub-optimal processing speed performance; this is indeed in tune with previous meta-analysis observation (Dickinson et al., 2007).

Overall, our study supports the evidence for specific information processing speed deficits in schizophrenia. Also, we could demonstrate the mechanistic possibilities of processing speed deficits (relational memory vs. visual scanning). The association between specific symptoms and processing speed deficits requires more systematic evaluation, as well as merits specific interventions included under cognitive remediation strategies. In addition, this fundamental cognitive deficit involving processing speed abnormalities needs large-scale systematic studies in the unaffected relatives to ascertain its endophenotype status in schizophrenia.
